# *Astragalus membranaceus* Injection Protects Retinal Ganglion Cells by Regulating the Nerve Growth Factor Signaling Pathway in Experimental Rat Traumatic Optic Neuropathy

**DOI:** 10.1155/2020/2429843

**Published:** 2020-12-18

**Authors:** Qiong Wu, Xinyi Gu, Xinyan Liu, Xiaoling Yan, Liang Liao, Jian Zhou

**Affiliations:** ^1^Department of Ophthalmology, Dongfang Hospital, Beijing University of Chinese Medicine, Beijing 100078, China; ^2^Department of Ophthalmology, Eastern District of Dongzhimen Hospital, Beijing University of Chinese Medicine, Beijing 101121, China

## Abstract

Activation of the nerve growth factor (NGF) signaling pathway is a potential method of treatment for retinal ganglion cell (RGC) loss due to traumatic optic neuropathy (TON). The present study aimed to explore the biological effects of injecting *Astragalus membranaceus* (*A. mem*) on RGCs in an experimental TON model. Adult male Wistar rats were randomly divided into three groups: sham-operated (SL), model (ML), and *A. mem* injection (AL). The left eyes of the rats were considered the experimental eyes, and the right eyes served as the controls. AL rats received daily intraperitoneal injections of *A. mem* (3 mL/kg), whereas ML and SL rats were administered the same volume of normal saline. The TON rat model was induced by optic nerve (ON) transverse quantitative traction. After two-week administration, the number of RGCs was determined using retrograde labeling with Fluoro-Gold. The protein levels of NGF, tyrosine kinase receptor A (TrkA), c-Jun N-terminal protein kinase (JNK), JNK phosphorylation (p-JNK), and nuclear factor kappa-B (NF-*κ*B) were assessed using western blotting. The levels of p75 neurotrophin receptor (p75^NTR^) and NF-*κ*B DNA binding were examined using real-time PCR and an electrophoretic mobility shift assay. In addition, the concentrations of JNK and p-JNK were assessed using an enzyme-linked immunosorbent assay. *Results*. The number of RGCs in ML was found to be significantly decreased (*P* < 0.01) relative to both AL and SL, together with the downregulation of NGF (*P* < 0.01), TrkA (*P* < 0.05), and NF-*κ*B (*P* < 0.01); upregulation of p75^NTR^ mRNA (*P* < 0.01); and increased protein levels of JNK (*P* < 0.05) and p-JNK (*P* < 0.05). Treatment using *A. mem* injection significantly preserved the density of RGCs in rats with experimental TON and markedly upregulated the proteins of NGF (*P* < 0.01), TrkA (*P* < 0.05), and NF-*κ*B (*P* < 0.01) and downregulated the mRNA level of p75^NTR^(*P* < 0.01), as well as the proteins of JNK (*P* < 0.05) and p-JNK (*P* < 0.01). Thus, *A. mem* injection could reduce RGC death in TON induced by ON transverse quantitative traction by stimulating the NGF signaling pathway.

## 1. Introduction

Traumatic optic neuropathy (TON) is a rare disease that can lead to severe irreversible visual impairment, which occurs usually secondary to orbital, ocular, head, or traumatic facial injuries. Research has shown that 79% of patients with TON are usually male, with a median age of 31 years [1]. Several studies have also reported that the most common causes for TON include motor vehicle and bike accidents, falls, and physical assault [2, 3]. According to the mechanism of causative injury, TON is categorized into direct and indirect TON [4, 5]. Direct TON usually occurs after a sharp trauma that causes direct damage to the optic nerve (ON) and is extremely rare thanks to the protection provided by the bony orbit [6]. In contrast, indirect TON occurs secondary to the damage caused by the transmitted forces after a concussive blow to the head or orbit, which occurs in 0.5–5% of patients with closed-head trauma [5, 7]. Several therapeutic approaches for TON exist in clinical practice, including intravenous corticosteroids and endoscopic surgical decompression [5]. However, the benefits of steroids are controversial, and surgery should be considered in patients with strict radiological evidence of compression [8, 9]. Therefore, a better potential therapeutic strategy for TON remains to be explored.

Generally, the ON comprises axons of retinal ganglion cells (RGCs) along with support cells [1]. RGCs are central nervous system (CNS) neurons that lack an endogenous regenerative capacity. Thus, after injury or under diseased conditions, damaged RGCs cannot be replaced and the damaged ON does not regenerate, leading to irreversible visual impairment [10–13]. The trauma caused by the impact can transmit to the nerve, particularly at the point where it enters the optic canal, consequently shearing the RGC axons [6, 14]. Hence, it is important to assess RGCs to develop relevant animal models of TON [15]. Studies have illustrated that RGC loss is related to the downregulation of nerve growth factor (NGF), a member of the neurotrophin family, which is produced during neuronal growth and differentiation and participates in growth, differentiation, survival, and apoptosis in the peripheral nervous system (PNS) and CNS [16]. NGF binds to both tyrosine kinase receptor A (TrkA) and P75^NTR^ [17]. NGF/TrkA signaling plays a crucial role in neuronal development, function, survival, and growth [18]. However, the affinity of p75^NTR^ for proNGF may induce cell survival through the nuclear factor kappa-B (NF-*κ*B) pathway and cell apoptosis through the activation of c-Jun N-terminal protein kinase (JNK) [19]. In addition, separate activation of either p75^NTR^ or TrkA is sufficient to lead to NF-*κ*B activation [20].

According to the basic theory of traditional Chinese medicine (TCM), Qi and blood are the basic material of human body as well as the material base of the internal organs and meridians. Therefore, the function of the eye depends on the nourishment of Qi and blood. The cereal essence can reach the eyes by the movement of Qi and then nourish the eyes. *Astragalus membranaceus* (*A. mem*) is a type of qi-tonifying drug in TCM, which could promote the circulation of Qi and blood in meridians. It can boost the essence reaching the eye so that the eye could have the visual function. Many researches have confirmed that Chinese herbal prescriptions containing *A. mem* are effective in treating optic atrophy (OA), ischemic retinopathy, glaucoma, and other eye diseases [21–23]. In addition, *A. mem* is one of the most commonly used TCM in the treatment of OA [24]. *A. mem* injection is a clinical preparation of *Astragalus* that is prepared using the following protocols: water extraction, alcohol precipitation, alkali addition, and adsorption of pigments by activated carbon. Studies have revealed through *in vitro* and *in vivo* experiments that *A. mem* protects RGC-5 cells against oxidative stress and apoptosis and boosts peripheral nerve regeneration [25, 26]. The extract of *A. mem* relieves nerve injury after cerebral ischemia by downregulating the apoptotic pathway of JNK signal transduction [27]. The total flavonoids of *A. mem* significantly suppress JNK in the mitogen-activated protein kinase (MAPK) pathway and the expression of nuclear NF-*κ*B p65 in the NF-*κ*B pathway [28]. Many studies have reported on the neuroprotective effect of *A. mem*; however, studies investigating the mechanism of *A. mem* in optic neuroprotection, particularly in TON, are few.

Therefore, in the current study, we aimed to investigate the effect of *A. mem* injection on RGC survival by quantification of RGCs, evaluating the changes of the protein levels of NGF, TrkA, JNK, JNK phosphorylation (p-JNK), and NF-*κ*B and the mRNA levels of P75^NTR^ in the retinal tissues of rats with experimental TON. We attempted to elucidate the molecular mechanism underlying the efficacy of *A. mem* injection as an effective treatment for TON.

## 2. Materials and Methods

### 2.1. Materials

Fluoro-Gold (FG) (Fluorochrome, Denver, CO, USA), *A. mem* injection (ChiaTai QingChunBao Pharmaceutical Co., Ltd., Hangzhou, China), rat RNAiso Plus kit (TaKaRa Biomedical Technology Co., Ltd., NO. D9108A), rat ReverTra Ace qPCR RT Kit (TOYOBO, Osaka, Japan), SYBR Premix Ex Taq (Takara Bio Inc, Kusatsu, Japan), marker (New England Biolabs, Inc., MA, USA), Rabbit anti-NGF antibody (ab52918, Abcam, Cambridge, UK), Rabbit anti-Pan TrkA (ab76291, Abcam, Cambridge, UK), Rabbit anti-SAPK/JNK (9258S, CST, MA, USA), Rabbit anti-P-SAPK/JNK (4668S, CST, MA, USA), Mouse anti-NF-*κ*B p65 (6956S, CST, MA, USA) and Rabbit anti-GAPDH (ab9485, Abcam, Cambridge, UK), Anti-mouse IgG (7076S, CST, MA, USA) and anti-rabbit IgG (ZB-2301, Zhongshan Golden Bridge Biotechnology Co., Ltd., Beijing, China), JNK1 ELISA kit (Elabscience Biotechnology Co., Ltd., Wuhan city, China), 6-0 polyester suture (Unik Surgical Sutures Mgf. Co., Ltd., Suzhou, China), transverse tensiometer (TOKYO SEIKI Co., Ltd., Tokyo, Japan). EMSA Probe Biotin Labeling Kit and Nuclear and Cytoplasmic Protein Extraction Kit were purchased from Beyotime (Beyotime Biotechnology Co., Ltd., Shanghai, China). Paraformaldehyde, bicinchoninic acid (BCA) Protein Assay kit, RIPA, 5% BSA Blocking Buffer, 1.5 M Tris-HCL (pH 8.8), SDS-PAGE Gel Kit, 1,2-Bis(dimethylamino)ethane, ammonium persulphate (APS), 1M Tris-HCL (pH 6.8), SDS-PAGE loading buffer, 4×(with DTT), 30%(29 : 1) acrylamide, WB Transfer Buffer (10×), Tris-Glycine Running Buffer (5×), TBE Buffer (5×), ECL Western Blotting Substrate, immobilon-PSQ PVDF (0.2 *µ*m), and hybond-N+ nylon membranes were all purchased from Solarbio (Beijing Solarbio Science Technology Co., Ltd., Beijing, China).

### 2.2. Animals

The animal care and experiments were approved by the Bioethics Committee of Dongfang Hospital of Beijing University of Chinese Medicine (No. 201618). Male Wistar rats, aged 6-7 weeks, were obtained from SPF Beijing Vital River Laboratory Animal Technology Co., Ltd. Rats were maintained in specific pathogen-free conditions at a temperature of 22°C, 12 : 12 hours of the light-dark cycle. All experiment protocols were executed according to the Guide for the Care and Use of Laboratory Animals and the Beijing Laboratory Animal Welfare Ethics Review Guidelines issued by the Ministry of Science and Technology of China.

### 2.3. Retrograde Labeling and Quantification of RGCs

The retrograde labeling of RGC with FG was performed one week before modeling. As a previous study described, 2 *μ*L of 5% FG was microinjected into each side of the superior colliculus of rat, which was fixed in a stereotaxic apparatus [29]. There were seven days of recovery for rats before further experiments. After two weeks of administration in each group, rats were anaesthetized by narcotic overdose and the eyes were enucleated. Following removal of the cornea and lens, the eye cup was fixed in 4% paraformaldehyde for 1 hour. Then, the retina was dissected from the choroid and flat-mounted on glass slides. The number of RGCs in the flat-mounted retina was counted under a fluorescence microscope (OLYMPUS IX81, excitation with length 438 nm). At least three retinal preparations were analyzed for each sample (*n* = 5 in each group). For quantification, twelve digital images were obtained per retina (superonasal, inferonasal, inferotemporal, and supratemporal regions of the flattened retina), corresponding to locations at 1/6, 1/2, and 5/6 of the retinal radius. The number of cells was counted in each field (200× magnification).

### 2.4. ON Transverse Quantitative Traction Surgery

The retina and retinal blood vessels of the fundus in rats were examined by an ophthalmoscope (Olympus Co., Ltd., Tokyo, Japan) before modeling. Rats were anaesthetized with 2 % pentobarbital sodium (50 mg/kg). Under a stereomicroscope, the rats were disinfected by iodine in the prone position. An incision was made in the lateral orbital margin of the left eye of a rat with conjunctival scissors and the oblique rectus muscle was detached under an operating microscope. The ON was exposed and isolated by blunt separation from the optic nerve sheath. The ON was wrapped and knotted with 6-0 polyester suture at a distance of 2 mm posterior to the globe. The circle length is equal to the circumference of the optic nerve cross-section. Care was taken to avoid being tightened around the ON. Transverse tensiometer was applied to connect with the end of the suture and was pulled perpendicular to the ON horizontally for 20 s with 0.10 N [30]. After the operation, the skin was sutured, and the wound was wiped with iodine and coated with erythromycin to resist inflammation.

### 2.5. Drug Administration

Sixty rats were divided into three groups using random numbers: a model group, an *A. mem* injection group, and a sham-operated group (20 rats in each group). In order to verify whether the operation except ON traction has an effect on NGF signaling pathway, we set up the sham-operated group. In rats in the sham-operated group, the ON of left eyes only were exposed and were sutured. In the model group and the *A. mem* injection group, the left eyes of rats were experimental eyes and the right eyes were healthy eyes for comparison. On the second day after the establishment of model, rats in *A. mem* injection group were administrated daily intraperitoneal injection (*IP*) of *A. mem* injection, which lasted two weeks. The previous research has reported that two-week treatment of *A. mem* injection has a neuroprotective effect on RGCs and regulates the expression of TrkB; therefore, the duration of treatment in this research was 2 weeks [31]. Each milliliter (ml) of *A. mem* injection contained 1 mg astragaloside (molecular weight: 784.97, molecular formula: C_41_H_68_O_14_). *A. mem* injection was injected intraperitoneally to rats with the dose of 3 ml/kg according to the research of Liu et al. which demonstrated that *A. mem* injection could inhibit neuronal apoptosis and downregulate the expression of JNK3 gene [32]. Rats in the other two groups received normal saline with same volume.

### 2.6. Quantitative Real-Time PCR Analysis

We first applied the RNAiso Plus kit to extract total RNA from retinas. The ReverTra Ace qPCR RT Kit was used for the first-strand cDNA synthesis. The qPCR was performed using SYBR Premix Ex Taq on an ABI 3900 Real-Time PCR system (Applied Biosystems, Foster City, CA, USA). All the reactions were repeated at least three times. The mRNA sequence of p75^NTR^ for rats was searched from the GenBank sequence database. The primers of p75^NTR^ were designed and composed by Life Technology (China) Co., Ltd. The primers used for p75^NTR^ were 5′-CCACCAGAGGGAGAGAAACTG-3′ and 5′- CCAGGTGTCACCATTGAGCAG-3′. The primers for *β*-actin were 5′-CTTCCTTCTTGGGTATGGAATCC-3′ and 5′-GGAGCAATGATCTTGATCTTCATG-3′. The comparative Ct method was used to perform relative quantification and results were shown in arbitrary units (AU). The expression level was calculated as 2−ΔCt and then compared with the model group while using the 2−ΔΔCt formula to calculate the fold change [33].

### 2.7. Western Blot Analysis

Retinal tissues were dissected and lysed in RIPA Lysis buffer. Then, the lysates were centrifuged at 14,000 g for 4 min at 4°C (Eppendorf China Ltd., Shanghai, China). The supernatants were collected. Protein concentrations were determined using BCA Protein Assay kit. According to the molecular weight of the target protein, samples were separated by 8%–15% SDS-PAGE gels and then transferred to nitrocellulose filter membranes. Next, the blots were blocked with 3% bovine serum albumin for 60 min and incubated in primary antibody which was used in the research: Rabbit anti-NGF antibody (1 : 1000), Rabbit anti-Pan TrkA (1 : 1000), Rabbit anti-SAPK/JNK (1 : 1000), Rabbit anti-P-SAPK/JNK (1 : 1000), Mouse anti-NF-*κ*B p65 (1 : 1000), and Rabbit anti-GAPDH (1 : 2500) overnight at 4°C. Then, they were incubated with corresponding horseradish peroxidase-conjugated secondary antibody: Anti-mouse IgG (1 : 2000) and anti-rabbit IgG (1 : 2000). Finally, chemiluminescent immunoreactive complexes were visualized via Gel Image Station (GeneModel Co., Ltd., Beijing, China). Band intensity was analyzed using Image J.

#### 2.7.1. Electrophoretic Mobility Shift Assays (EMSA)

To assess the DNA binding activity of NF-*κ*B, EMSA was performed by the EMSA Probe Biotin Labeling Kit. The nuclear protein was extracted from the retina with the Nuclear and Cytoplasmic Protein Extraction Kit according to the instructions. The protein content was assessed using the BCA protein assay kit. The sequence of the NF-*κ*B oligonucleotide probe was 5′-AGTTGAGGGACTTTCCCAGGC-3′. The mixture was separated by electrophoresis on a 4.0% polyacrylamide gel in 0.5X Tris-borate-EDTA buffer. Finally, blots were transferred onto N+ nylon membranes to detect DNA-protein complexes by the imaging system.

#### 2.7.2. Enzyme-Linked Immunosorbent Assay (ELISA)

The retinal tissue and PBS were mixed at 1 : 9 and were fully grounded on ice. Then, they were centrifugated at 5,000 rpm for 10 min at 4°C, and the supernatant was taken for testing. The JNK1 ELISA kit was used to measure the concentration of JNK according to the manufacturer's protocol. Sandwich-ELISA principle was used in this kit. The microplate in this kit was pre-coated with a specific antibody of Rat JNK1. Samples and standards were added to the microplate wells and combined with the antibody. After ninety-minute incubation, the unbound substances were washed away. Then, 100 *μ*l enzyme-linked antibody was added to each microplate well. After one-hour incubation at 37°C, free components were removed. Next, we added the substrate solution to each well for 30 min incubation. The stop solution was added to terminate the enzyme-substrate reaction. The optical density (OD) was measured spectrophotometrically at an absorbance of 450 nm. The OD value was read, and the concentration of JNK1 was calculated based on the standard curve.

### 2.8. Statistical Analysis

The data in the density of RGC labeled with FG are presented as mean ± standard deviation (SD). Other data are shown as means ± standard error of the mean (SEM). One-way analysis of variance (ANOVA) was performed using SPSS 21.0 software (IBM SPSS, Shanghai, China). Comparison tests between groups were performed using Dunnett's post hoc analysis. It was considered statistically significant if *P* < 0.05.

## 3. Results

### 3.1. *A. mem* Injection Promotes RGC Survival after ON Transverse Quantitative Traction

**Figure 1:**
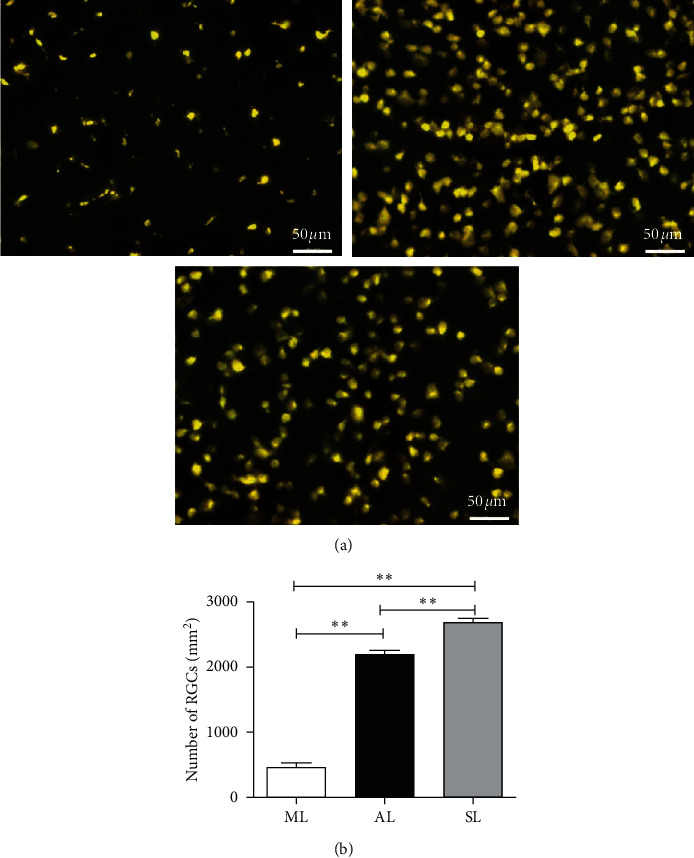
Retrogradely labeling RGC with Fluoro-Gold (FG). (a) Survival of retinal ganglion cell (RGC) two weeks after ON transverse quantitative traction. RGC was retrogradely labeled with FG in model group (i), AI group (ii), and sham-operated group (iii). Magnification: 200×, scale bar: 50 *μ*m. (b) Quantification of retrogradely labeled RGC. The number of RGC in AL and SL was significantly higher than that of ML. Data are presented as mean ± SD (*n* = 5). ^*∗∗*^*P* < 0.01, versus ML. ML, left eyes of the model group; AL, left eyes of *A. mem* injection group; SL, left eyes of the sham-operated group.

To determine the ability of *A. mem* injection to alleviate RGC loss, we labeled RGCs with Fluoro-Gold (FG) and estimated the density of RGCs in flat-mounted retinas 14 days after ON transverse quantitative traction.  Figure 1(a) shows representative micrographs of FG-labeled cells. As shown in Figures 1(a) and 1(b), at two weeks after modeling, the number of labeled RGCs markedly declined in the model group (ML; left eyes) and *A. mem* injection group (AL; left eyes) when compared to sham-operated group (SL; left eyes) (*F*_1_ = 55.89, *P*_1_ < 0.01; *F*_2_ = 13.01, *P*_1_ < 0.01). Furthermore, significantly more RGCs were found in eyes treated with *A. mem* injection compared to ML (*F* = 38.66, *P* < 0.01*P*). These morphological results revealed that *A. mem* injection reduces RGC death after insult and contributes to retinal neuroprotection.

### 3.2. *A. mem* Injection Activates the NGF/TrkA Signaling Pathway

**Figure 2:**
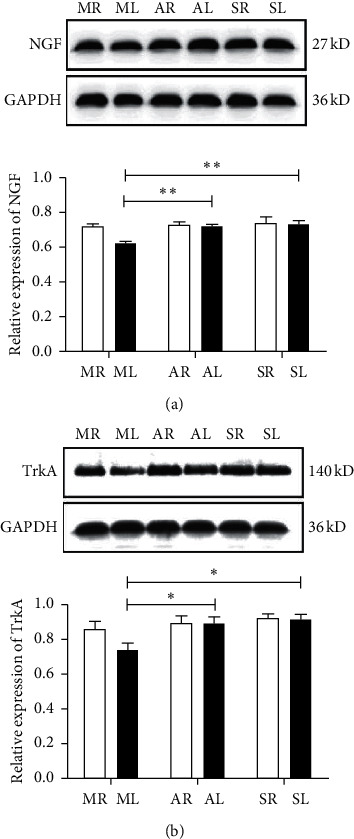
NGF and TrkA relative expression protein levels in the RGCs at two weeks after ON transverse quantitative traction in rats. (a, b) The protein levels of NGF and TrkA by western blot. Expression was normalized to the GAPDH expression level. Data are presented as mean ± SEM (*n* = 4). ^*∗*^*P* < 0.01 and ^*∗∗*^*P* < 0.01, versus ML. MR, the right eyes of model group; ML, the left eyes of the model group; AR, the right eyes of *A. mem* injection group; AL, the left eyes of *A. mem* injection group; SR, the right eyes of the sham-operated group; SL, the left eyes of the sham-operated group.

To verify whether *A. mem* injection boosts the expression of NGF and TrkA, western blotting was performed to test their protein levels. Figures 2(a) and 2(b) show that there was a significant increase in the levels of NGF and TrkA in AL compared to those observed in ML (*F*_1_ = 24.70, *P*_1_ < 0.01; *F*_2_ = 6.72, *P*_2_ < 0.05). Meanwhile, it was found that SL exhibited the same trend as that of AL (*F*_1_ = 18.55, *P*_1_ < 0.01; *F*_2_ = 12.24, *P*_2_ < 0.05). These results suggest that the neuroprotective role of *A. mem* injection is related to the upregulation of NGF and the subsequent downstream signaling of TrkA.

### 3.3. *A. mem* Injection Regulates the Expression of p75NTR and NF-*κ*B

**Figure 3:**
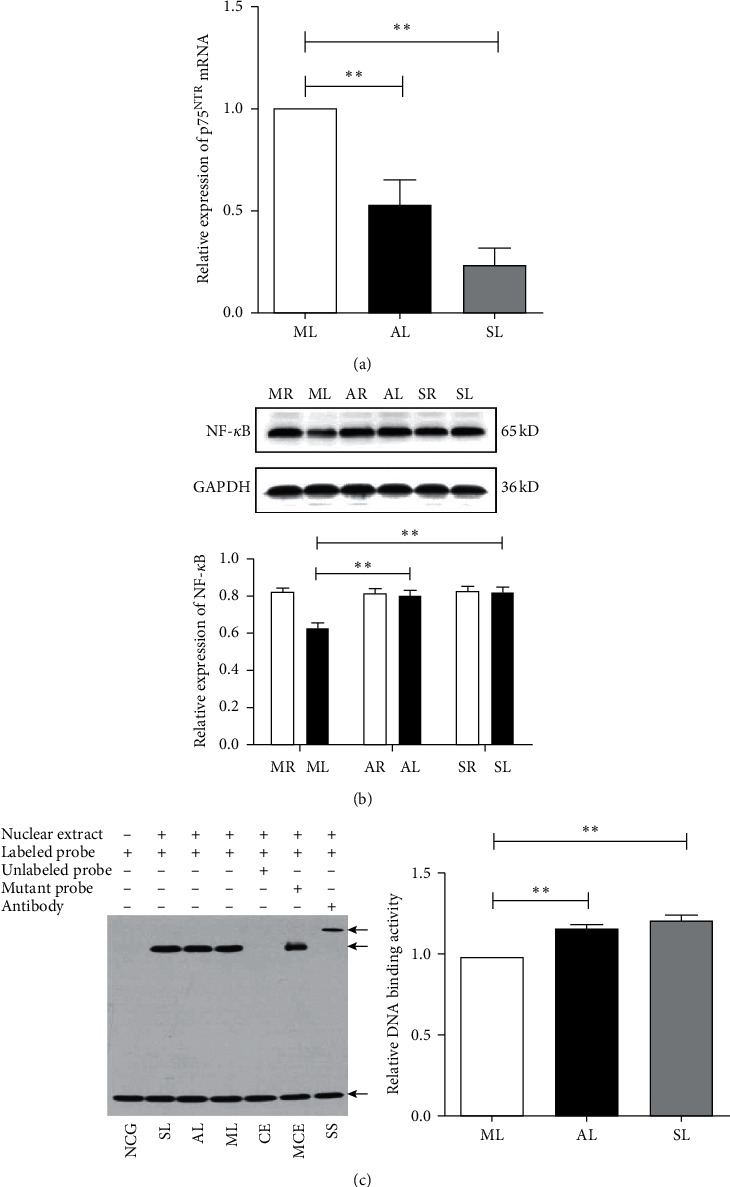
Effects of *A. mem* injection on p75NTR and NF-*κ*B levels in the RGCs at two weeks after ON transverse quantitative traction in rats. (a) Real-time PCR for p75NTR mRNA. Expression was normalized to ML expression level. Data are presented as mean ± SEM (*n* = 3). ^*∗∗*^*P* < 0.01, versus ML. (b) The protein levels of NF-*κ*B by western blot. Expression was normalized to the GAPDH expression level. Data are presented as mean ± SEM (*n* = 4). ^*∗∗*^*P* < 0.01, versus ML. (c) Quantitative analyses of NF-*κ*B activation from EMSA results. The three arrows from top to bottom represent super-shift, DNA-protein complex, and free-probe, respectively. Activities were normalized to ML activity level. Data are presented as mean ± SEM (*n* = 3). ^*∗∗*^*P* < 0.01, versus ML. NF-*κ*B, nuclear factor-*κ*B; EMSA, electrophoretic mobility shift assay; NCG, negative control reaction; SL, the left eyes of sham-operated group; AL, the left eyes of *A. mem* injection group; ML, the left eyes of model group; CE, competitive experiment; MCE, competitive experiment of mutant probe; SS, super-shift; MR, the right eyes of model group; AR, the right eyes of *A. mem* injection group; SR, the right eyes of sham-operated group.

We evaluated whether *A. mem* injection affects the expression of p75^NTR^ and NF-*κ*B after ON transverse quantitative traction. First, we checked the mRNA level of p75^NTR^ using real-time PCR to explore the effect of *A. mem* injection on the expression of p75^NTR^ in the rats' retinas. As shown in Figure 3(a), the mRNA level of p75^NTR^ was found to be higher in ML than in SL and AL (*F*_1_ = 81.73, *P*_1_ < 0.01; *F*_2_ = 14.87, *P*_2_ < 0.01). Next, the expression of NF-*κ*B was detected using western blot analysis.  Figure 3(b) shows that the level of NF-*κ*B in SL was significantly higher than that in ML (*F* = 18.38, *P* < 0.01). Moreover, after treatment using *A. mem* injection for two weeks, a marked increase was observed in the level of NF-*κ*B in AL (*F* = 16.05, *P* < 0.01). In addition, the DNA-binding activity of NF-*κ*B was analyzed using an electrophoretic mobility shift assay. The results presented in Figure 3(c) indicate that the activity of NF-*κ*B was significantly lower in ML than in AL and SL (*F*_1_ = 2.86, *P*_1_ < 0.05; *F*_2_ = 3.98, *P*_2_ < 0.05). Collectively, our data illustrated that treatment using *A. mem* injection significantly downregulates the expression of p75^NTR^ and boosts the expression of NF-*κ*B in retinas with ON transverse quantitative traction.

### 3.4. *A. mem* Injection Inhibits the Expression of p-JNK and JNK

**Figure 4:**
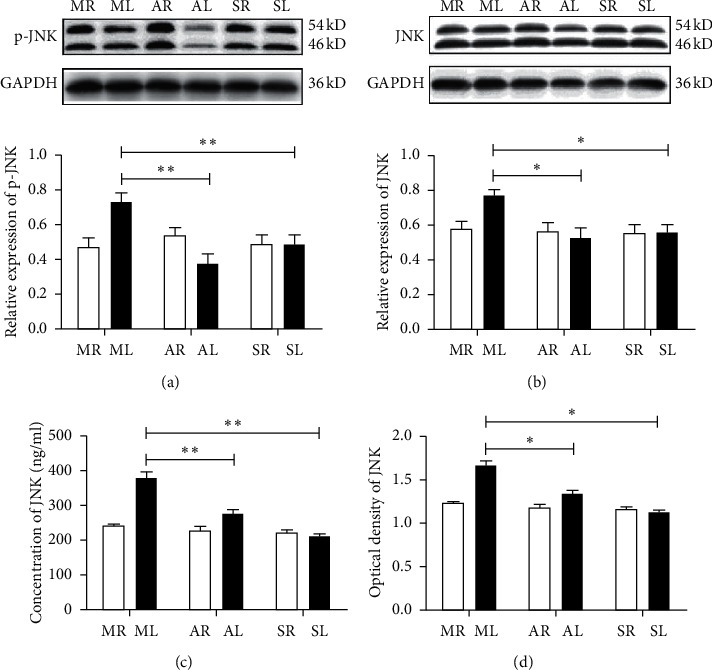
Effects of AI on the p-JNK and JNK levels in the RGCs at two weeks after ON transverse quantitative traction in rats. (a, b) The protein levels of p-JNK and JNK by western blot. Expression was normalized to the GAPDH expression level. Data are presented as mean ± SEM (*n* = 4). ^*∗*^*P* < 0.05, ^*∗∗*^*P* < 0.05, versus ML. (c and d) The concentration and optical density at 450 nm (OD450) of JNK by ELISA test. Data are presented as mean ± SEM (*n* = 4). ^*∗∗*^*P* < 0.01, versus ML. MR, the right eyes of the model group; ML, the left eyes of the model group; AR, the right eyes of *A. mem* injection group; AL, the left eyes of *A. mem* injection group; SR, the right eyes of the sham-operated group; SL, the left eyes of the sham-operated group.

To assess the expression of p-JNK and JNK in the retina, western blot analysis was performed. As shown in Figures 4(a) and 4(b), the total protein levels of p-JNK and JNK in ML were significantly higher than in SL (*F*_1_ = 8.83, *P*_1_ < 0.05; *F*_2_ = 12.06, *P*_2_ < 0.05). Furthermore, the levels of p-JNK and JNK in AL significantly decreased after a two-week treatment using *A. mem* injection (*F*_1_ = 18.42, *P*_1_ < 0.01; *F*_2_ = 11.27, *P*_2_ < 0.05). Moreover, enzyme-linked immunosorbent assay was performed to determine the concentration of JNK in the retina. The optical density (OD) was measured spectrophotometrically at a wavelength of 450 nm (OD_450_) and was found to be proportional to the concentration of JNK. Figures 4(c) and 4(d) demonstrate that both the JNK concentration and OD_450_ value in ML were significantly higher than those in SL (*F*_1_ = 70.64, *P*_1_ < 0.01; *F*_2_ = 68.42, *P*_2_ < 0.01). Meanwhile, the concentration and OD_450_ value of JNK in AL were found to be significantly lower than those in ML (*F*_1_ = 20.40, *P*_1_ < 0.01; *F*_2_ = 20.00, *P*_2_ < 0.05). Collectively, our data indicated that treatment using *A. mem* injection in rats after ON transverse quantitative traction suppresses the expression of JNK and p-JNK, which is closely associated with alleviating the apoptosis of RGCs.

## 4. Discussion

TON leads to RGC death and axon degeneration in the ON. Thus, evaluating RGC survival and function is crucial in developing relevant animal models of TON [34]. Three classical rat models are widely applied in studying retinal and ON injury: ON transection, ON crush (ONC), and ocular blast injury [35]. ON transection and ONC are both models of direct TON: they are considered easy to perform and directly damage the nerve by cutting or crushing [36]. Transection models produce complete injury of the ON and interruption of all axons so that it is limited in terms of studying ON protection and treatment [36, 37]. In ONC model, injury could be either complete or incomplete, depending on the magnitude and duration of the force applied [35]. The drawback of the ONC model though is that it cannot calculate the force applied on the forceps to obtain crush injury [36]. The ocular blast model is a noninvasive model studied in research on indirect TON. The main disadvantage of the model is the serious anterior and posterior segment damage accompanying the ON trauma and leading to a high mortality rate, ranging from 24% to 46% [38]. In this research, we developed a new model of TON, namely, ON transverse quantitative traction. In this model, the exposed ON was wrapped and knotted with 6-0 polyester suture equal to the circumference of the ON's cross section. A transverse tensiometer was used to connect the end of the suture and was pulled perpendicular to the ON horizontally for 20 s with a force of 0.10 N. Before carrying out this research, we tested different durations (5, 10, 15, 20, 25, and 30 s) with 0.1 N of traction to investigate the survival rate of RGCs, and we observed that the RGCs rapidly degenerated three days after modeling when the duration was over 15 s. Five days following modeling, the number of RGCs decreased by half with a duration of 20 s. Therefore, traction for 20 s with a force of 0.10 N was deemed suitable. Previous research on model validation showed that 1, 3, 7, and 14 days after the transverse quantitative traction of the ON, the number of RGCs progressively decreased even below that in normal rats. In addition, the survival rates of RGCs at 1, 3, 7, and 14 days after modeling were 78.94%, 60.07%, 38.92%, and 17.31%, respectively [30]. Therefore, in this model, there is no need for the axons to be interrupted completely, and the operator can monitor the magnitude and duration of the force applied precisely.

Retinal degeneration can directly or indirectly result in RGC dysfunction or loss [39, 40]. Therefore, assessing RGCs plays a vital role in evaluating retinal degeneration, particularly in TON. In addition, reliable and accurate RGC labeling is essential for evaluating the efficacy of neuroprotective therapies. Almost 98% of RGCs in rats project to their axons directly to the contralateral superior colliculus (SC) [41]. The SC, as the axon terminals of RGCs in rats, can be injected using retrograde fluorescent tracer FG, which then absorbs the FG through an active or passive mechanism and transfers it to the soma and dendrites through axonal flow [42, 43]. Then, only RGCs stained with FG are seen and counted precisely under a fluorescence microscope. Therefore, it is assumed that any axonal dysfunction can directly influence the number of labeled RGCs [44]. It has been reported in some studies that this FC labeling can persist in the RGCs for two months without apparent fading or leaking [45]. Therefore, it is concluded that retrograde tracing of FG is a sensitive and effective technique for the identification and quantitation of RGCs [46]. In this research, a significant decrease was observed in the number of RGCs labeled with FG after modeling, and it is assumed that treatment with *A. mem* injection could promote RGC survival.

Purified *A. mem* injection includes 18 active ingredients which are shown in Table 1 [47, 48]. Among these ingredients, astragaloside IV is considered to be the most effective in neuroprotection. There is plenty of literature on the relationship between astragaloside IV and regeneration in the CNS and PNS [49, 50]. It has also been reported that astragaloside I could promote axonal maturation [51]. However, the neuroprotective effects of the 16 remaining components in *A. mem* injection remain to be further studied. All in all, it is assumed that all 18 ingredients in *A. mem* injection are collectively efficacious for the treatment of rat TON.

NGF, the first neurotrophin discovered and the most widely investigated one, plays a crucial role in neuronal growth, differentiation, survival, and regeneration [52]. This neurotrophin can be produced and utilized by RGCs in the retina. Research has demonstrated that NGF protects RGCs against injury induced by ON transection, retinal ischemia-reperfusion, ischemic injury, and glaucoma [53–57]. We showed earlier that the protein level of NGF is significantly downregulated in the retina of rats after ON transverse quantitative traction. However, the results demonstrated that *A. mem* injection increased the expression of NGF in the injured retina.

NGF binds to two distinct cell surface receptors: TrkA and p75^NTR^. TrkA is selective to NGF binding and is considered the high-affinity catalytic receptor for NGF [58], and it also mediates the main effects of NGF. As illustrated in previous research, the binding between NGF and the TrkA receptor can mediate many processes, such as the survival and regeneration of neurons, the growth of axons, the expression of neurotransmitters, and the avoidance of programmed cell death [59]. In this study, it was found that the TrkA protein level decreased in the retina after the insult, but it increased again with the 14-day administration of *A. mem* injection. This result is in line with previous research [59–61] and verifies that *A. mem* injection plays a role in restraining RGC death by activating NGF/TrkA.

However, the effect of p75^NTR^ on RGCs still needs to be further explored. p75^NTR^, a member of the tumor necrosis factor (TNF) receptor superfamily, is the only neurotrophin receptor with a significant binding affinity for all neurotrophins, including NGF, brain-derived neurotrophic factor, neurotrophin-3, and neurotrophin-4/5 [62]. Studies have reported that the binding of NGF to p75^NTR^ can initiate an intracellular pathway, which is similar to activation by TNF and Fas receptors, produces ceramide, and thus directly induces apoptosis [62–64]. We also found out that the mRNA level of p75^NTR^ was significantly upregulated in the retinas of rats after ON transverse quantitative traction. Moreover, a significant decrease in p75^NTR^ occurred after treatment using *A. mem* injection. Therefore, it is assumed that *A. mem* injection has the potential to reduce p75^NTR^ in the retinas of rats after ON transverse quantitative traction.

In addition, p75^NTR^ can activate the signaling pathway for both the activation of JNK and translocation of NF-*κ*B, which are associated with cellular survival [19]. JNK is a member of the MAPK family, which is a phosphorylation-dependent signaling system known to be a significant pro-death signaling pathway in damaged neurons and also belongs to a phosphorylation-dependent signaling system [65]. Research has shown that, in RGCs, the JNK signaling pathway can be activated after insult induced by axonal injury, retinal ischemia, excitotoxicity, or glaucoma [66–69]. Additionally, it was found that JNK inhibitors promote the survival of RGCs [70, 71]. NF-*κ*B was first discovered and characterized over 30 years ago as a crucial inducible transcription factor that regulates immune and inflammatory reactions, cell growth and survival, and cancer biology [72–74]. As revealed in several studies, in the nervous system, NF-*κ*B appears to exert either a proapoptotic or an antiapoptotic effect, depending on the cell type and circumstances [75]. Research *in vitro* has revealed that the activation of NF-*κ*B in neurons can prevent excitotoxic and metabolic injury related to the pathogenesis of traumatic injury [20]. In addition, the stimulation of NF-*κ*B could boost the survival of neurons, whereas the stimulation of NF-*κ*B in glial cells may induce the generation of neurotoxins [20, 76, 77]. Furthermore, research has illustrated that separate activation of either p75^NTR^ or TrkA is sufficient to lead to NF-*κ*B activation and that both pathways can interact downstream, which has a potential impact on the expression of NF-*κ*B [20]. Therefore, to explore the effect of JNK- and NF-*κ*B-dependent pathways on RGCs, we examined the levels of JNK, p-JNK, and NF-*κ*B, and the results showed that the protein levels of p-JNK and JNK were higher in ML than in SL. However, no significant difference was observed between AL and SL. On the contrary, in the present study, the binding activity of NF-*κ*B in the nucleus, as well as the protein level of NF-*κ*B in ML, was found to be lower than that in SL and AL, suggesting that *A. mem* injection could inhibit the death of RGCs by suppressing the JNK signaling pathway and boosting the NF-*κ*B signaling pathway in the retina. Although this research uncovered many findings, the reason why NF-*κ*B changes in the retina after modeling and treatment remains to be further explored, to determine whether these changes were induced either through separate activation of p75^NTR^ or TrkA or through an interaction between them.

## 5. Conclusions

Our results revealed that *A. mem* injection significantly alleviates the apoptosis of RGCs by activating NGF/TrkA, inhibiting the expression of P75^NTR^ and JNK, and increasing the protein level of NF-*κ*B. In conclusion, we suggest that *A. mem* injection could be regarded as a promising treatment for TON in the future, which is associated with the initiation of the NGF signaling pathway.

## Figures and Tables

**Table 1:** 18 active ingredients in purified *A. mem* injection.

No.	Name of ingredients
1	Pratensein 7-*O*-glucopyranoside
2	Calycosin-7-*O*-*β*-D-glucoside
3	4′-Hydroxyisoflavone-7-*O*-*β*-D-glucoside
4	6″-Acetyl-calycosin-7-D-glucoside
5	Pterocarpane-3-glucoside
6	Methylnissolin-3-*O*-glucoside
7	Pratensein
8	Isomucronulatol
9	Isorhamnetin
10	Isorhamnetin-3-O-gentiobioside
11	Medicarpin
12	Lariciresinol B9
13	Astragalin
14	Astragaloside I
15	Astragaloside IV
16	Astragaloside V
17	Astragaloside VI
18	Astragaloside VII

## Data Availability

The data used to support the findings of this study are available from the corresponding author upon request.
